# Folic Acid Represses Hypoxia-Induced Inflammation in THP-1 Cells through Inhibition of the PI3K/Akt/HIF-1α Pathway

**DOI:** 10.1371/journal.pone.0151553

**Published:** 2016-03-14

**Authors:** Xiaoyan Huang, Zhiying He, Xinwei Jiang, Mengjun Hou, Zhihong Tang, Xiaozhou Zhen, Yuming Liang, Jing Ma

**Affiliations:** 1 Department of Nutrition, School of Public Health, Sun Yat-Sen University, Guangzhou, P. R. China; 2 Experimental and Teaching Center for public health, School of Public Health, Sun Yat-Sen University, Guangzhou, P. R. China; University of Thessaly, Faculty of Medicine, GREECE

## Abstract

Though hypoxia has been implicated as a cause of inflammation, the underlying mechanism is not well understood. Folic acid has been shown to provide protection against oxidative stress and inflammation in patients with cardiovascular disease and various models approximating insult to tissue via inflammation. It has been reported that hypoxia-induced inflammation is associated with oxidative stress, upregulation of hypoxia-inducible factor 1-alpha (HIF-1α), and production of pro-inflammatory molecules. Whether folic acid protects human monocytic cells (THP-1 cells) against hypoxia-induced damage, however, remains unknown. We used THP-1 cells to establish a hypoxia-induced cellular injury model. Pretreating THP-1 cells with folic acid attenuated hypoxia-induced inflammatory responses, including a decrease in protein and mRNA levels of interleukin (IL)-1β and tumor necrosis factor-alpha (TNF-α), coupled with increased levels of IL-10. Folic acid also reduced hypoxia-induced Akt phosphorylation and decreased nuclear accumulation of HIF-1α protein. Both LY294002 (a selective inhibitor of phosphatidyl inositol-3 kinase, PI3K) and KC7F2 (a HIF-1α inhibitor) reduced levels of hypoxia-induced inflammatory cytokines. We also found that insulin (an Akt activator) and dimethyloxallyl glycine (DMOG, a HIF-1α activator) induced over-expression of inflammatory cytokines, which could be blocked by folic acid. Taken together, these findings demonstrate how folic acid attenuates the hypoxia-induced inflammatory responses of THP-1 cells through inhibition of the PI3K/Akt/HIF-1α pathway.

## Introduction

Hypoxia is defined as a condition of reduced oxygen tension (pO_2_), and it is not only relevant to the oxygen tension present in the atmosphere, it is also an intrinsic component of human pathology and physiology [1, 2]. Hypoxia is an important component of the pathogenesis of vascular and inflammatory diseases [3]. Monocytes serve a key function during the body’s innate immune defense, and also are the cells that possess the ability to regulate the immune system through immune stimulation and suppression [4]. Monocytes are able to differentiate into elicited macrophages within tissues. During the onset of inflammation, wound healing, and other diseases, symptoms of disease are usually accompanied by extensive extravasation by monocytes. Hypoxic conditions reportedly have a profound effect on a wide range of monocyte properties in vitro. Some examples of the parameters affected by hypoxia are the expression of cell surface markers, cell viability, cellular migration, cellular adhesion, and cytokine secretion [4]. This evidence suggests hypoxia may play a role in the process of inflammation.

Hypoxia-inducible factor (HIF) is a heterodimeric transcription factor composed of an α and a β subunit. There are three oxygen-sensitive alpha subunits (HIF-1α, HIF-2α and HIF-3α), which are induced rapidly in response to hypoxia [5]. The β-subunit, also known as ARNT, is also up-regulated in response to hypoxia in a cell-specific manner [6]. Current research indicates that HIF-1α is the HIF factor most critical for the response to hypoxia; it mediates cell proliferation, cellular survival, angiogenesis, cell migration, and cell invasion, determines levels HIF-1 activity, and is strictly regulated by cellular oxygen tension [7]. Under normal oxygen conditions, HIF-1α is modified at two conserved prolines and hydroxylated by prolyl hydroxylases (PHDs). This leads to HIF-1α polyubiquitylation via a specific von Hippel-Lindau-E3 ligase complex, and HIF-1α is subsequently degraded by proteasomes [8, 9]. Recently, HIF-1 has been reported to be activated during the immune response, playing an important role in inflammation [10, 11]. Previous studies have shown that cytokines induced from macrophages, such as inducible nitric oxide synthase (iNOS), TNF-α, IL-1, and IL-6, are up-regulated under hypoxic conditions at both the gene and protein levels [10, 12–14]. This work suggests that HIF-1α plays an integrative signaling role during hypoxic conditions and inflammation. With the exception of HIF-1α, hypoxia adaptive vascular endothelial growth factors (VEGF) are up-regulated in response to hypoxia. The hypoxia response element of VEGF is bound by the global oxygen sensor HIF under hypoxic conditions, resulting in the up-regulation of VEGF genes [15].

The PI3K/Akt signaling pathway is important for controlling HIF-1α protein levels during hypoxia. The pathway works by increasing HIF-1α protein synthesis [16]. Many stimuli are able to activate Akt, including hypoxia. During hypoxic conditions, Akt is the target of PI3K in the inner leaflet of the plasma membrane. Then, Akt is phosphorylated on the Ser-473 residue (in the regulatory domain) and Thr-308 residue (in the catalytic domain) by a phosphoinositide-dependent protein kinase. This cascade leads to enzymatic activation of Akt [17]. The involvement of this pathway in the regulation of HIF-1α activity, however, remains controversial.

Folic acid, also known as vitamin B_9_, is required for optimal health, growth, and development [18, 19]. Folic acid is the precursor for tetrahydrofolate, and is necessary for many biological processes. Particularly, folic acid is necessary for one-carbon transfer reactions, nucleic acid synthesis, and methionine regeneration [20]. A variety of clinical abnormalities are related to folic acid deficiency, like carcinogenesis and cardiovascular disease [21]. Folic acid supplementation has been reported to be a promising approach for prevention and treatment of cardiovascular diseases associated with hyperhomocysteinemia or other risk factors [22, 23]. It is well-known that pathogenesis of cancer and cardiovascular diseases, including atherosclerosis, involves inflammation as a crucial component [24, 25]. We propose folic acid plays a protective role in inflammation-related diseases due to its anti-inflammatory effects. As evidence, folic acid has been found to exert anti-inflammatory effects in healthy, overweight subjects as well as in patients with inflammatory bowel diseases [26, 27]. Several studies have confirmed the anti-inflammatory effects of folic acid in vivo [26, 27]. However, the effects of folic acid on monocytes and the underlying mechanisms are rarely studied. In addition, the signal transduction pathways affected by folic acid remain elusive.

In this study, we conducted experiments to test the hypothesis that treatment with folic acid would attenuate the hypoxia-induced inflammatory response in THP-1 cells. We also investigated the mechanistic role of folic acid in the HIF-1α and PI3K/Akt signaling pathways.

## Materials and Methods

### Reagents and antibodies

Folic acid, dimethyl sulfoxide (DMSO) and insulin were purchased from Sigma–Aldrich (St. Louis, MO, USA). LY294002 was purchased from Merck Biosciences (New Jersey, USA). KC7F2 was purchased from Millipore (Billerica, MA, USA). Dimethyloxallyl glycine (DMOG) was purchased from Cayman Chemical (Ann Arbor, MI, USA). RPMI 1640, fetal bovine serum, antibiotics, and all other cell culture reagents were purchased from GIBCO/BRL Life Technologies (Grand Island, NY). The HIF-1α antibody was purchased from BD Biosciences (New Jersey, USA). The phospho-Akt (Ser 473) and Akt antibodies were purchased from Cell Signaling Technology (Danvers, USA). β-actin and Lamin B1 antibodies were obtained from Proteintech Group Inc (Chicago, IL, USA).

### Cell culture and viability assay

#### Cell culture

Human monocyte THP-1 cells were generously provided by Professor Wenhua Ling (Department of Nutrition, School of Public Health, Sun Yat-sen University, Guangzhou, China). THP-1 cells were cultured in RPMI 1640-complete medium that was supplemented with 10% fetal bovine serum, 50 units/mL penicillin, and 50 mg/mL streptomycin sulfate under normal oxidative conditions (5% CO_2_, 95% air).

#### Exposure to hypoxia

Prior to induction of hypoxia, all cells were cultured in serum-free media for 24 hours. Cells were then exposed to hypoxia conditions or normal oxidative conditions for an indicated amount of time. Before exposure to hypoxic conditions, THP-1 cells were placed in a humidified, airtight incubator with inflow valves. Then, the air was pumped out and replaced with a hypoxic gas mixture consisting of 1% O_2_, 5% CO_2_, and 94% N_2_. The airtight incubator was kept at 37°C for 24 hours. In contrast, cells exposed to normal oxidative conditions were cultured at 37°C in an incubator with 5% CO_2_ and 95% air until harvest.

#### Cell viability determination

3-(4,5-dimethylthiazol-2-yl)-2,5-diphenyltetrazo -lium bromide (MTT) assay (Sigma–Aldrich, Deisenhofen, Germany) was conducted in order to investigate the toxic effects of folic acid on THP-1 cells. 2×10^5^ cells/well were seeded into 96-well plates and incubated with free-serum medium for 24 hours. Cells were then treated with different concentrations of folic acid (0–40 μg/mL) and cultured for 24 hours. After incubation, 20 μL MTT (5 mg/mL) was added to each well and cells incubated at 37°C for 4 hours. 100 μL of a SDS-isobutanol-HCl solution consisting of 10% SDS, 5% isobutanol, and 12 μM HCl were added to each well and the cells were incubated overnight. Absorption was measured at 570 nm using a microplate reader (Bio-Rad, San Diego, CA). Six wells per dose were counted in four independent experiments. The readings were normalized to account for background absorbance by subtracting the readings of a blank treatment from each reading.

### Western blot analysis

To prepare the nuclear and the cytoplasmic fractions, cells were lysed using a nuclear and cytoplasmic protein extraction kit (Beyotime Biotechnology, Shanghai, China) according to the manufacturer’s instructions. Total cellular protein was extracted from cultured cells. Western blot analysis was performed as previously described [28]. Typical 30–75 mg total protein was used to perform the blots, and protein was incubated with primary antibodies specific to phospho-Akt (1:2000), Akt (1:1000), HIF-1α (1:1500), Lamin B1 (1:10000) and β-actin (1:10000). The blots were then incubated with HRP-conjugated secondary antibodies (1:2000–4000) and visualized using ECL reagent (Thermo fisher, Rockford, USA).

### ELISA analysis

Cells culture supernatants were collected and measured by ELISA (eBioscience, San Diego, CA, USA) to detect levels of IL-10, IL-1β, and TNF-α. ELISA was performed in accordance with the manufacturer’s manual.

### RNA preparation and qRT-PCR

Total RNA was extracted from cells using Trizol reagent (Invitrogen, Carlsbad, CA, USA) according to the manufacturer's instructions. 1 μg of total RNA was reverse-transcribed into cDNA using Transcriptor cDNA Synth (Roche, Basel, CH) as described in the manufacturer’s protocol. The primer sequences used are listed in Table 1. qRT-PCR was performed using Fast Start Universal SYBR Green Master Mix (Roche, Basel, CH) on the Vii7 system (ABI, Carlsbad, CA, USA). Quantification was done using a two-step real-time PCR protocol. The protocol consisted of a denaturation step at 95°C for 10 min, 40 cycles at 95°C for 15 s, and a subsequent step at 60°C for 1 min. Data analysis was done using the comparative Ct method (2^ΔΔCt^). For each clone, experiments were performed in triplicate.

**Table 1:** Primer Sequences Used for qRT-PCR.

Genes	Primer	Sequence
TNF-α	Forward	5’-CAG CAA GGA CAG CAG AGG A-3’
	Reverse	5’-CCG TGG GTC AGT ATG TGA GA-3’
IL-1β	Forward	5’-TCT TCG ACA CAT GGG ATA ACG A-3’
	Reverse	5’-TCC CGG AGC GTG CAG TT- 3’
IL-10	Forward	5’-GAC TTT AAG GGT TAC CTG GGT TG-3’
	Reverse	5’-TCA CAT GCG CCT TGA TGT CTG-3’
VEGF	Forward	5’-ATG ACG AGG GCC TGG AGT GTG-3’
	Reverse	5’-CCT ATG TGC TGG CCT TGG TGA G- 3’
β-actin	Forward	5’-CCT GGC ACC CAG CAC AAT-3’
	Reverse	5’-GGG CCG GAC TCG TCA TAC- 3’

### Imunofluorescence staining

Cells were treated with folic acid under the hypoxia conditions described above. After treatment, cytospin preparations were obtained on polylysine-coated slides (Boster, Wuhan, China) by centrifuging the cells for 5 minutes and fixing the cells for 10 minutes using 4% paraformaldehyde. Slides were washed 3 times with PBS for 5 minutes each time, and then treated with 0.1% Triton X-100 in PBS for 10 minutes at 4°C. This step was performed in order to permeabilize the cell membranes. Then, blocking was performed in 5% goat serum-PBS for 15 min. Then, they were incubated with anti-HIF-1α (1:100 dilution) overnight. After washing, as mentioned above, Andy flour 555-conjugated goat anti-mouse IgG (1:200 dilution, GeneCopoeia, Rockville, MD, USA) was used to immunolabel HIF-1α for 45 minutes at room temperature. The nuclei were then counterstained with 4',6-diamidino-2-phenylindole (DAPI). Cells were analyzed using a confocal laser-scanning microscope (Leica, Germany). Fluorescence intensity was quantified using Image Pro Plus 6.0 software (NIH Bethesda, MD, USA).

### Statistics

All experiments were performed at least three times. All data are expressed as means ± SEM. Significant differences between groups were assessed by performing a one-way ANOVA using SPSS 19.0 software (SPSS Inc, Chicago, IL, USA). Differences between groups were compared using the least significant difference (LSD) test. Individual *P* values are given in the figure legends. A value of *P*<0.05 was considered statistically significant.

## Results

### Assessment of folic acid toxicity to cells

In order to exclude the possibility that any phenotype was due to the cytotoxicity of folic acid, the viability of cultured THP-1 cells was assayed after a 24 hours incubation at different folic acid concentrations (0–40 μg/mL). Viability analysis was performed by using the MTT assay. THP-1 cellular viability was not significantly affected by folic acid during treatment at 0.4–40 μg/mL (S1 Fig).

### Folic acid regulated hypoxia-induced inflammatory cytokines secretion and mRNA expression in THP-1 cells

**Fig 1:**
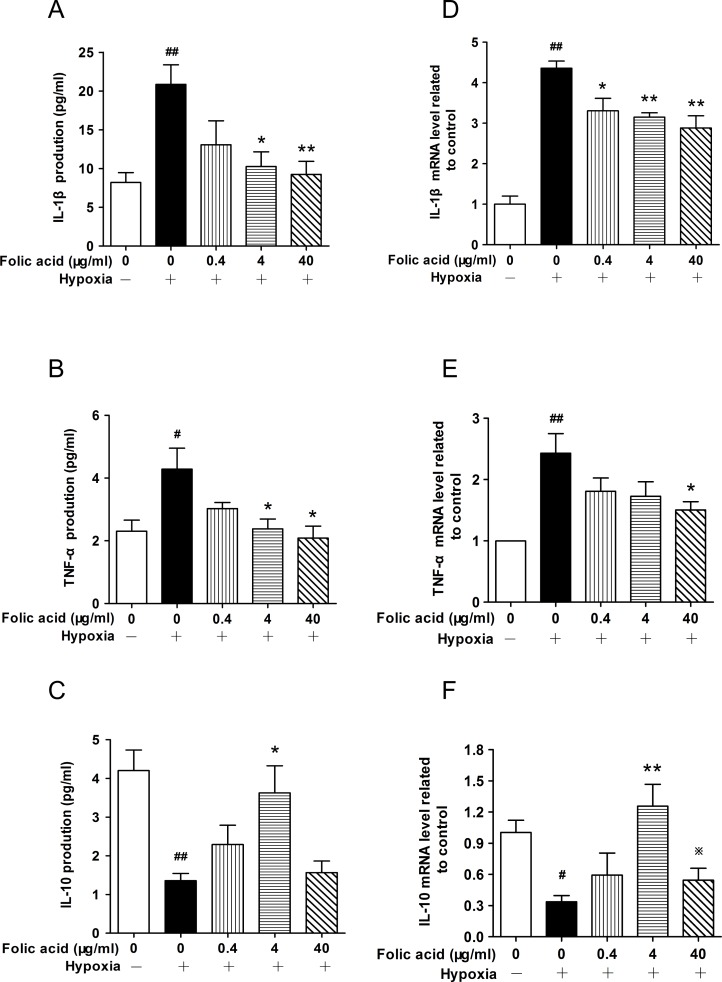
Folic acid regulation of hypoxia-induced IL-1β, TNF-α, and IL-10 secretion and mRNA expression. THP-1 cells were exposed to 1% O_2_ for 24 hours in the absence or presence of folic acid pretreatment at the indicated concentrations for 30 minutes. ELISA was performed to detect the levels of IL-1β (A), TNF-α (B), and IL-10 (C) in cell supernatants. qRT-PCR was performed to measure mRNA levels of IL-1β (D), TNF-α (E), and IL-10 (F). Data are shown as the mean ± SEM of six independent experiments. ^#^*P*<0.05, ^##^*P*<0.01 compared with the control group under normal oxidative conditions. *P<0.05, **P<0.01 compared with the hypoxia group.^※^*P*<0.05 compared with the group treated with folic acid at 4 μg/mL.

IL-1β, IL-10 and TNF-α are cytokines typically secreted from inflammatory cells which can positively regulate the progression of inflammation. In this study, we detected the effects of folic acid treatment and hypoxia on THP-1 cells. The cells were pretreated with folic acid (0–40 μg/mL) for 30 minutes, then exposed to hypoxic conditions for 24 hours to cause inflammatory reactions. As shown in Fig 1A–1C, hypoxia increased the levels of IL-1β and TNF-α 3-fold and 2-fold respectively when compared with normoxia. In contrast, hypoxia decreased levels of IL-10 nearly 3-fold. Pre-treatment with folic acid inhibited IL-1β and TNF-α secretion and enhanced IL-10 secretion in a dose-dependent manner. Interestingly, a high dose of folic acid (40 μg/mL) decreased IL-10 levels. We also used qRT-PCR to assay the expression of IL-1β (Fig 1D), TNF-α (Fig 1E), and IL-10 (Fig 1F) mRNA and found that the gene expression followed a pattern similar to protein expression after folic acid treatment and exposure to hypoxic conditions. These data suggest that folic acid exerts an anti-inflammatory effect through reduced IL-1β and TNF-α protein and mRNA levels and increased the levels of IL-10 mRNA and protein.

### Folic acid inhibited hypoxia-induced p-Akt, HIF-1α protein accumulation, and VEGF mRNA expression, but not HIF-1α mRNA levels

**Fig 2:**
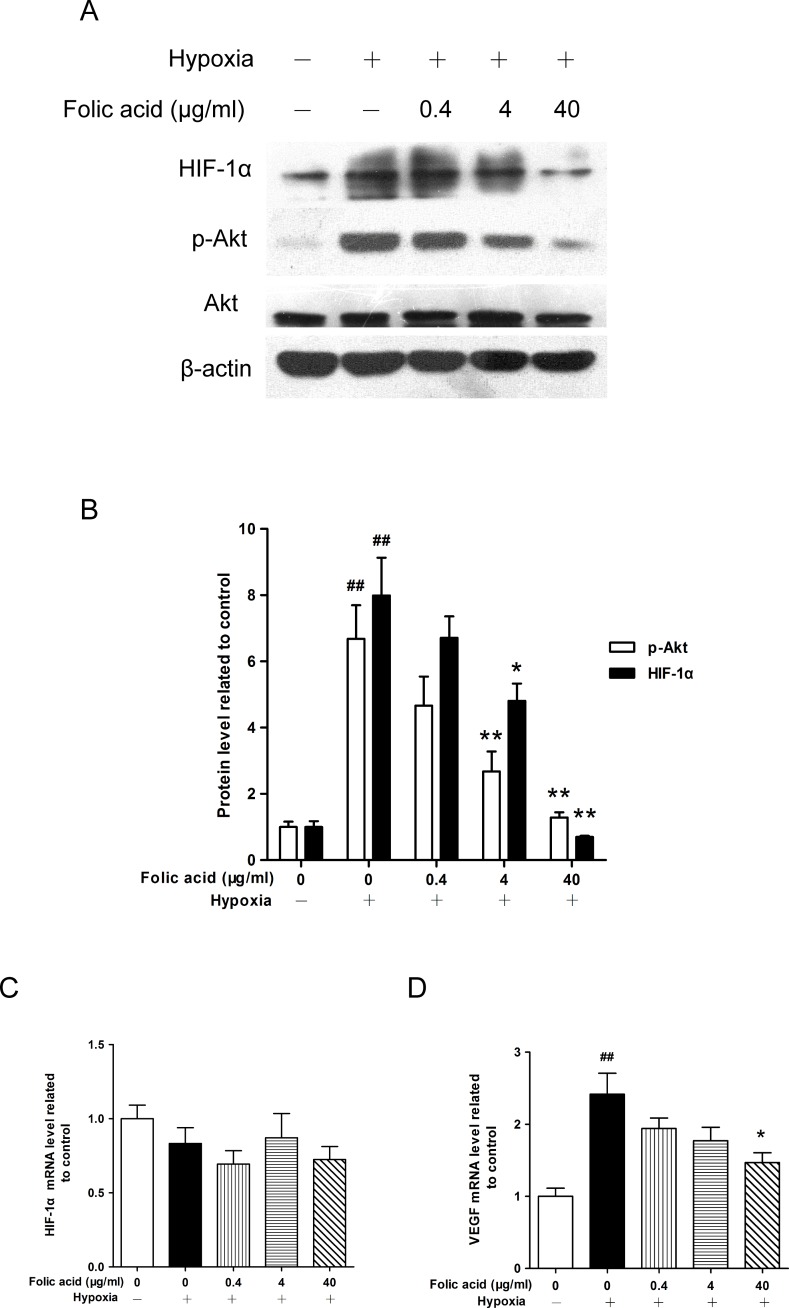
Effect of folic acid on p-Akt induction, HIF-1α mRNA and protein levels, and VEGF mRNA expression under hypoxic conditions. THP-1 cells were exposed to 1% O_2_ for 24 hours in the absence or presence of folic acid pretreatment at the indicated concentrations for 30 minutes. (A) Cell lysates were subjected to Western blot analysis using p-Akt, Akt, and HIF-1α specific antibodies. (B) The intensity of the protein bands from three typical experiments was quantified using Quantity One software. (C) HIF-1α mRNA levels were measured by qRT-PCR. (D) VEGF mRNA levels were measured by qRT-PCR. Data are shown as the mean ± SEM of three independent experiments. ^#^*P*<0.05, ^##^*P*<0.01 compared to the control group under normal oxidative conditions. *P<0.05, **P<0.01 compared to the hypoxia group.

To investigate the underlying mechanism by which folic acid pretreatment affects inflammatory cytokine production, we examined the HIF-1α and PI3K/Akt signaling pathway. As shown in Fig 2A, compared to the control cells cultured under normal oxidative conditions, HIF-1α and phospho-Akt^(Ser473)^ (p-Akt) protein levels were significantly up-regulated, after 24 hours of exposure to hypoxic conditions of 1% O_2_. After analyzing the gray value, HIF-1α and p-Akt protein levels increased by approximately 9-fold and 4-fold, respectively (Fig 2B). In contrast, total Akt protein levels were not affected. In THP-1 cells, folic acid treatment down-regulated HIF-1α protein levels under hypoxic conditions in a dose-dependent manner. This phenotype was observed in parallel with a decrease in p-Akt protein levels. Interestingly, compared to normal oxidative conditions, HIF-1α mRNA levels were not significantly changed in hypoxia-induced THP-1 cells, regardless of folic acid pretreatment (Fig 2C). We found that hypoxia increased VEGF mRNA expression, while folic acid treatment decreased VEGF mRNA levels in a dose-dependent manner (Fig 2D).

### Folic acid repressed nuclear HIF-1α accumulation under hypoxic conditions in a dose-dependent manner

**Fig 3:**
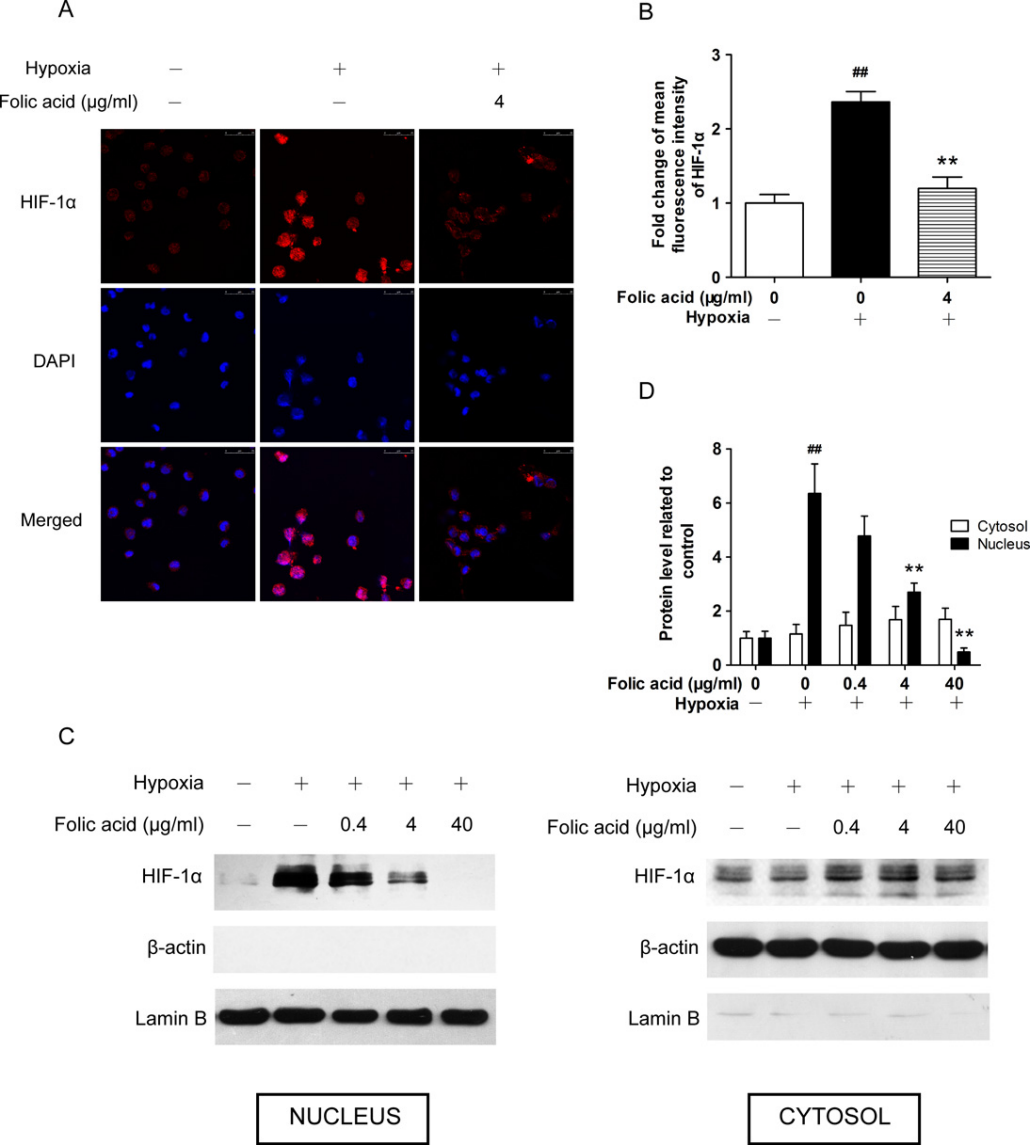
Effect of folic acid on HIF-1α intracellular accumulation under hypoxia. THP-1 cells were exposed to 1% O_2_ for 24 hours in the absence or presence of folic acid pretreatment at 4 μg/mL for 30 minutes. (A) THP-1 cells were incubated with anti-HIF-1α overnight and then stained with Andy flour 555-conjugated goat anti-mouse IgG as described in the methods section. Nuclei were stained with DAPI. Immunofluorescence was analyzed by confocal microscopy. Magnification bars = 25 μm. (B) The mean fluorescence intensity of the cells from three typical experiments was quantified using Image Pro Plus software. (C) Folic acid enhanced HIF-1α nuclear accumulation. HIF-1α was detected by Western blot analysis of the nuclear (left panel) and cytoplasmic (right panel) protein fractions extracted from THP-1 cells. (D) The intensity of the protein bands of three typical experiments was quantified using Quantity One software. Data are shown as the mean ± SEM. ^#^*P*<0.05, ^##^*P*<0.01 compared to the control group under normal oxidative conditions. *P<0.05, **P<0.01 compared with the hypoxia group.

HIF-1α accumulation under hypoxic condition occurs mainly in the nucleus [29], where it regulates its hypoxia-induced target genes. We investigated the effects of folic acid on the intracellular accumulation of HIF-1α in THP-1 cells by confocal laser-scanning microscopy (Fig 3A). Under normal oxidative conditions, HIF-1α was scarcely visible in THP-1 cells. Under hypoxic conditions, a strong nuclear signal of HIF-1α was detected. After pretreatment with folic acid, however, the HIF-1α signal became weaker (*P*<0.01, Fig 3B).

We also measured nuclear and cytoplasmic HIF-1α protein levels by Western blot, using β-actin as a cytoplasmic marker and Lamin B1 as a nuclear marker. The presence of HIF-1α was enhanced in the nuclear fraction after exposure to hypoxic conditions for 24 hours (Fig 3C and 3D). Treatment of cells with increasing concentrations of folic acid resulted in reduced nuclear HIF-1α levels. Meanwhile, cytoplasmic HIF-1α levels slightly changed without significance (Fig 3C and 3D). Thus, folic acid treatment inhibited the accumulation of HIF-1α protein in the cell nucleus in a dose-dependent manner.

### Folic acid promoted to cytoprotection through the PI3K/Akt/HIF-1α pathway under hypoxic conditions

**Fig 4:**
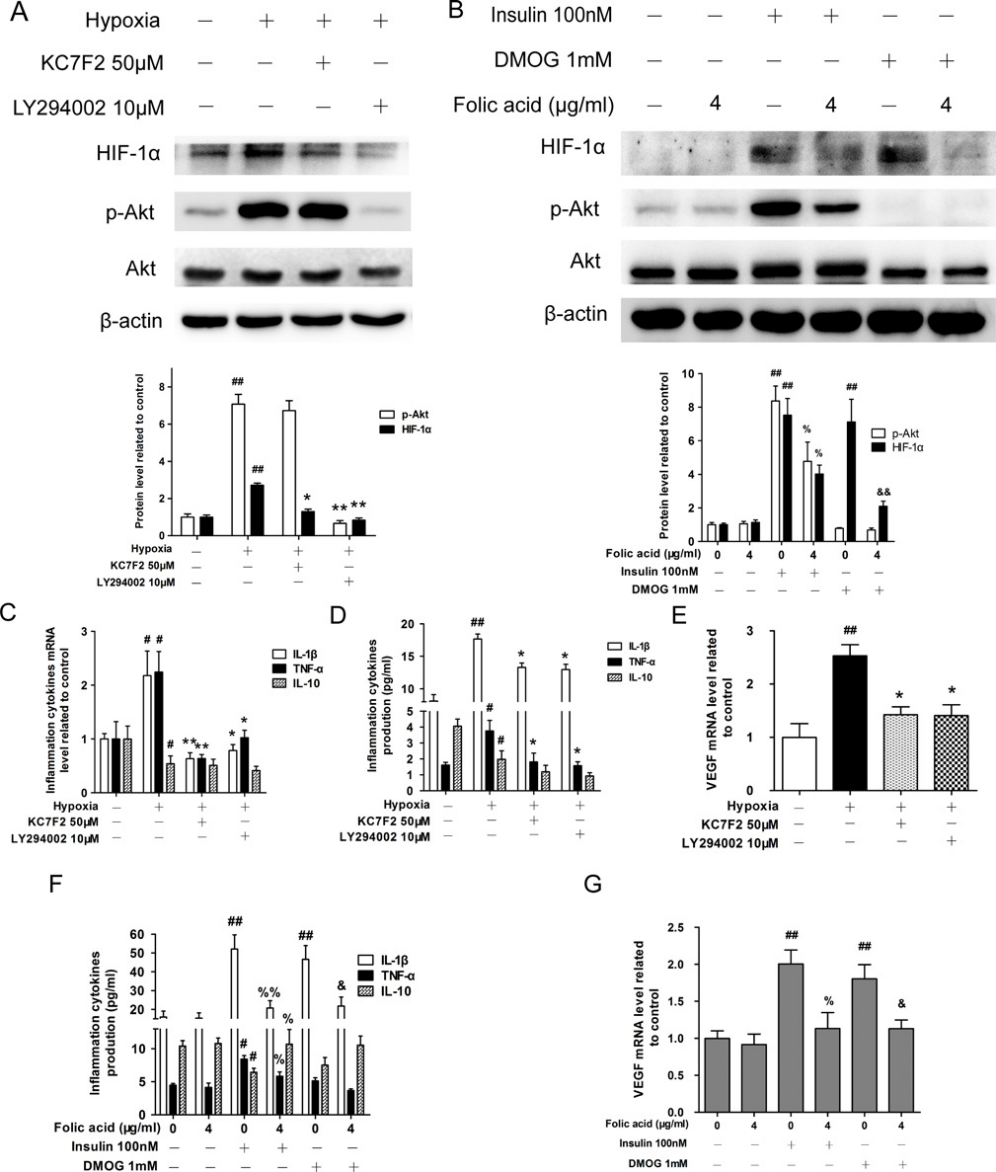
Folic acid suppresses hypoxia-induced expression of inflammatory cytokines via the inhibition of p-Akt and HIF-1α. Before cells were cultured under hypoxic conditions for 24 hours, THP-1 cells were pretreated with 50 μM KC7F2 or 10 μM LY294002 or 100nM insulin or 1mM DMOG with or without folic acid for 30 minutes. (A, B) Cell lysates were subjected to Western blot analysis using p-Akt, Akt, and HIF-1α specific antibodies. The intensity of the protein bands of three typical experiments was quantified using Quantity One software. IL-1β, TNF-α and IL-10 mRNA expressions and protein secretions (C, D, F), so as to VEGF mRNA (E, G) were detected by qRT-PCR or ELISA, respectively. Data are shown as the mean ± SEM of five independent experiments. ^#^*P*<0.05, ^##^*P*<0.01 compared with the control group under normal oxidative conditions. *P<0.05, **P<0.01 compared with the hypoxia group. ^%^*P*<0.05, ^%%^*P*<0.01 compared with the insulin group. ^&^*P*<0.05, ^&&^*P*<0.01 compared with the DMOG group.

LY294002 is a typical PI3K inhibitor [30] and KC7F2 is a specific inhibitor of HIF-1α [31]. To investigate the role of the PI3K/Akt/HIF-1α pathway in hypoxia-induced inflammation and cytokine secretion, THP-1 cells were pretreated with either LY294002 or KC7F2 for 30 minutes before subjection to hypoxic conditions. In THP-1 cells, the PI3K pathway was activated under hypoxic conditions, as evidenced by high levels of p-Akt. Pretreatment with LY294002 attenuated p-Akt, HIF-1α, IL-1β, and TNF-α protein levels (Fig 4A & 4C and 4D), while IL-10 levels were down-regulated in response to LY294002 treatment (Fig 4C and 4D). Pretreatment with KC7F2 before hypoxia had similar inhibitory effects on cytokine production: levels of HIF-1α were reduced, but p-Akt levels were not affected (Fig 4A). We also observed that both LY294002 and KC7F2 down-regulated hypoxia-induced VEGF mRNA expression (Fig 4E). These data indicate that signaling through the PI3K/Akt/HIF-1α pathway is required for hypoxia-induced secretion of pro-inflammatory cytokines.

To further probe the mechanisms of cytoprotection against hypoxia by folic acid, we utilized insulin as an agonist of Akt and the prolyl hydroxylase inhibitor DMOG as an agonist of HIF. Insulin stimulation resulted in a 8-fold up-regulation of p-Akt and a 7-fold increase in HIF-1α (Fig 4B), as well as increased IL-1β and TNF-α protein levels (Fig 4F). Surprisingly, insulin treatment down-regulated IL-10 protein levels. Pre-treatment with 4 μg/mL folic acid abolished the activation of p-Akt, the increased expression of HIF-1α and inflammatory cytokines. The effects of DMOG on cytokine induction were similar to those observed following insulin pretreatment (Fig 4F). After incubation with DMOG levels of HIF-1α were increased, but levels of p-Akt were not affected (Fig 4B). Further, we observed that folic acid blocked the increase in HIF-1α levels and the expression of inflammatory cytokines induced by DMOG (Fig 4F). In addition, VEGF mRNA was up-regulated by both insulin and DMOG treatment, but was down-regulated by the addition of folic acid (Fig 4G).

## Discussion

Hypoxia is characteristic of inflammatory diseases such as atherosclerosis [24] and cancer [25]. Previous research has indicated that hypoxia stimulates the expression of inflammatory cytokines in macrophages and endothelial cells in vitro [32, 33]. It is known that hypoxia is a distinguishing feature of high altitudes. It has been reported that circulating IL-6, IL-1 receptor antagonist (IL-1RA), and C-reactive protein (CRP) are up-regulated in response to hypoxic conditions at high altitudes, even in healthy people [34]. In addition, hypoxia-induced inflammatory cytokines may contribute to the development of high-altitude pulmonary oedema (HAPO) and other cardiovascular diseases [34, 35]. We assumed that folic acid intake would decrease the release of inflammatory cytokines in inhabitants living at high altitudes, because our previous research found that folic acid exhibited anti-inflammatory effects on LPS-induced NO, TNF-α, and IL-1β production via repression of MAPKs and NF-κB activation in RAW 264.7 cells [36]. In this study, we exposed THP-1 cells to hypoxic conditions, to establish an cellular model of the inflammation that is present in inhabitants of high-altitude environments.

The secretion of inflammatory cytokines from monocyte cells can be induced by a variety of exogenous conditions including hypoxia, and TNF-α plays a key role in the induction and perpetuation of inflammation under hypoxic conditions. It up-regulates other pro-inflammatory cytokines and endothelial adhesion molecules, which, in turn, enhance the recruitment of leukocytes to the site of inflammation. This cascade leads to autoimmune reactions by activating T cells and macrophages [37]. Similarly, IL-1β is another important pro-inflammatory cytokine that induces the expression of a variety of genes and the synthesis of several proteins. These genes and proteins induce acute and chronic inflammatory changes [38]. IL-10 is recognized for its ability to inhibit activation and effector function of T cells, monocytes, and macrophages. The routine function of IL-10 is to limit, and ultimately terminate inflammatory responses [39]. A significant reduction of the level of IL-10 was found as the oxygen tension was reduced from 21% to 2% (from normoxia to hypoxia) from placental explants, while secretion of TNF-α showed an increase with decreased oxygen tension [40]. Moreover, hypoxia also up-regulated the mRNA and protein levels of IL-1β [41]. In our study, we found the presence of IL-1β and TNF-α in cell culture supernatants was enhanced by hypoxia, whereas the presence of IL-10 was inhibited. We also demonstrated that folic acid represses hypoxia-induced inflammatory cytokines on mRNA expressions (IL-1β, TNF-α), while augmenting the expression and secretion of IL-10 in adaptive doses (0.4–4 μg/mL) in THP-1 cells. In contrast, IL-10 was inhibited by high doses of folic acid (40 μg/mL). It is possible that the pathway controling the regulation of secretion of IL-10 was repressed at high doses of folic acid during pretreatment, but the underlying mechanism is still unclear. To our knowledge, this is the first evidence that appropriate doses of folic acid are capable of inhibiting hypoxia-induced inflammatory cytokine production and secretion in monocyte cells.

We observed a decrease in HIF-1α accumulation after treatment with folic acid, which coincided with impaired Akt phosphorylation under hypoxic conditions in THP-1 cells. HIF-1α is a key transcription factor known to control adaptation to low-oxygen conditions. Our study illustrated how folic acid treatment of THP-1 cells leads to HIF-1α down-regulation in a dose-dependent manner. HIF-1α protein increased in THP-1 cells during hypoxia, whereas its mRNA expression levels decreased with no statistical significance. The results were similar to a previous study indicating that HIF-1α protein accumulates in total cell lysates, while HIF-1α mRNA levels decrease in hypoxia-induced THP-1 cells, rather than in differentiated macrophages [42]. The group that performed the previous study hypothesized that undifferentiated THP-1 cells may have higher basal HIF-1α mRNA levels due to their leukaemic origin. However, HIF-1α mRNA was not affected by a range of concentrations of folic acid in hypoxia-induced THP-1 cells. This finding is consistent with the previous study showing that mRNA levels of HIF-1α were not affected by evodiamine [43]. It is conceivable that folic acid reduced HIF-1α levels through increased degradation of HIF-1α protein, but the mechanism was undefined. Moreover, in folic acid-pretreated cells, hypoxia-induced VEGF was significantly attenuated.

We also observed that the decrease in total HIF-1α protein in whole cells was due primarily to changes in nucleus after treatment with folic acid. During hypoxia HIF-1α is stabilized and forms dimers with HIF-1β in nucleus [44]. This leads to increased expression of HIF-1 target genes under hypoxic conditions, such as CAIX [45] and VEGF [46]. Importin α/β is a classical nuclear-cytoplasmatic transporter involved in shuttling macromolecules between the cytoplasm and the nucleus, and nuclear accumulation of HIF-1α appears to be dependent on importin α/β [44]. Other members of the nuclear transport receptor family, such as importins 4 and 7, are also involved in the nuclear import of HIF-1α [47].

The PI3K/Akt signaling pathway is a crucial regulator of cell proliferation and angiogenesis. This pathway plays a major role in regulating HIF-1α protein accumulation through activation of the epidermal growth factor receptor (EGFR) and PI3K. This occurs in tandem with inactivation of the tumour suppressor phosphatase and tensin homolog, deleted on chromosome ten (PTEN) [48]. It has been reported that HIF-1α accumulation under hypoxia contributes to release of inflammatory cytokines. This has been observed for IL-1β, IL-8, MCP-1, and other cytokines [49–51]. Under hypoxia, the inflammatory process is significantly reduced in a functional knockout of HIF-1α [10]. We observed that hypoxia-induced HIF-1α accumulation was attenuated when cells were treated with the PI3K inhibitor LY294002 or HIF-1α inhibitor KC7F2, an effect similar to the effect of folic acid on hypoxia-induced cytokines.

We hypothesized that folic acid regulates hypoxia-induced inflammatory cytokines through the PI3K/Akt/HIF-1α pathway.I Insulin (an Akt inhibitor) and DMOG (an HIF-1α inhibitor) both increased IL-1β and TNF-α secretion, but decreased IL-10 secretion. Moreover, folic acid attenuated IL-1β and TNF-α expression in the presense of insulin or DMOG. In addition, VEGF, a downstream target of HIF-1α, showed a pattern of expression similar to these pro-inflammtory cytokines. Combined with data from previous investigations, our results demonstrate that folic acid regulates IL-1β, TNF-α, and IL-10 expression by interfering with the PI3K/Akt/HIF-1α signaling pathway in a dose-dependent manner under hypoxia.

**Fig 5:**
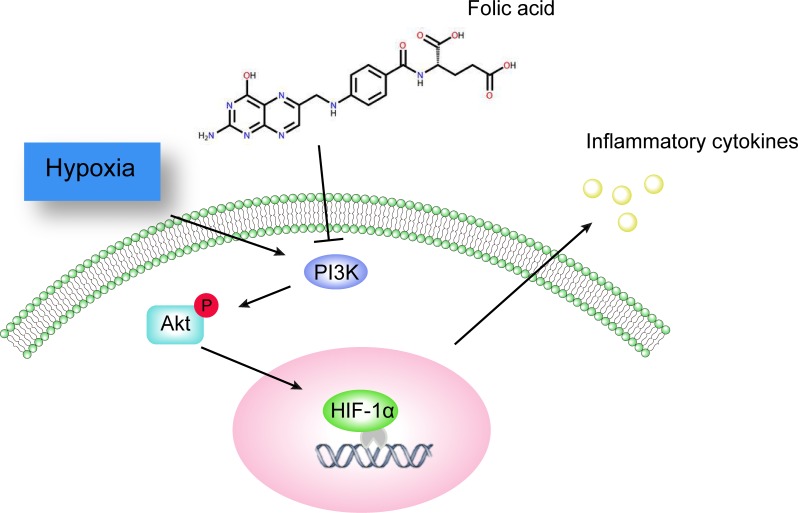
Schematic model of the possible mechanism of inflammatory cytokines down-regulation by folic acid. Folic acid represses inflammatory cytokine secretion and mRNA expression by inhibiting the PI3K/Akt/HIF-1α pathway under hypoxic conditions.

In conclusion, to our knowledge, this is the first report revealing how folic acid exerts anti-inflammatory effects by interfering with the PI3K/Akt/HIF-1α pathway in THP-1 cells under hypoxia (Fig 5). Our study implies that folic acid, as a universal vitamin, could provide potent protection against hypoxia-associated inflammatory injury. However, further studies are required, to fully understand the mechanism by which folic acid becomes intracellular and acts upon the PI3K/Akt pathway.

## Supporting Information

S1 Fig Assessment of folic acid cytotoxicity in THP-1 cells. The cytotoxic effects of folic acid in THP-1 cells were assessed using the MTT assay. Data are presented as mean ± SEM from four independent experiments.
(TIF)
