# Supplementary figures and images for: Folic Acid Represses Hypoxia-Induced Inflammation in THP-1 Cells through Inhibition of the PI3K/Akt/HIF-1α Pathway
Source: PLoS One. 2016 Mar 14;11(3):e0151553. doi: 10.1371/journal.pone.0151553 (PMC4790958; doi:10.1371/journal.pone.0151553)

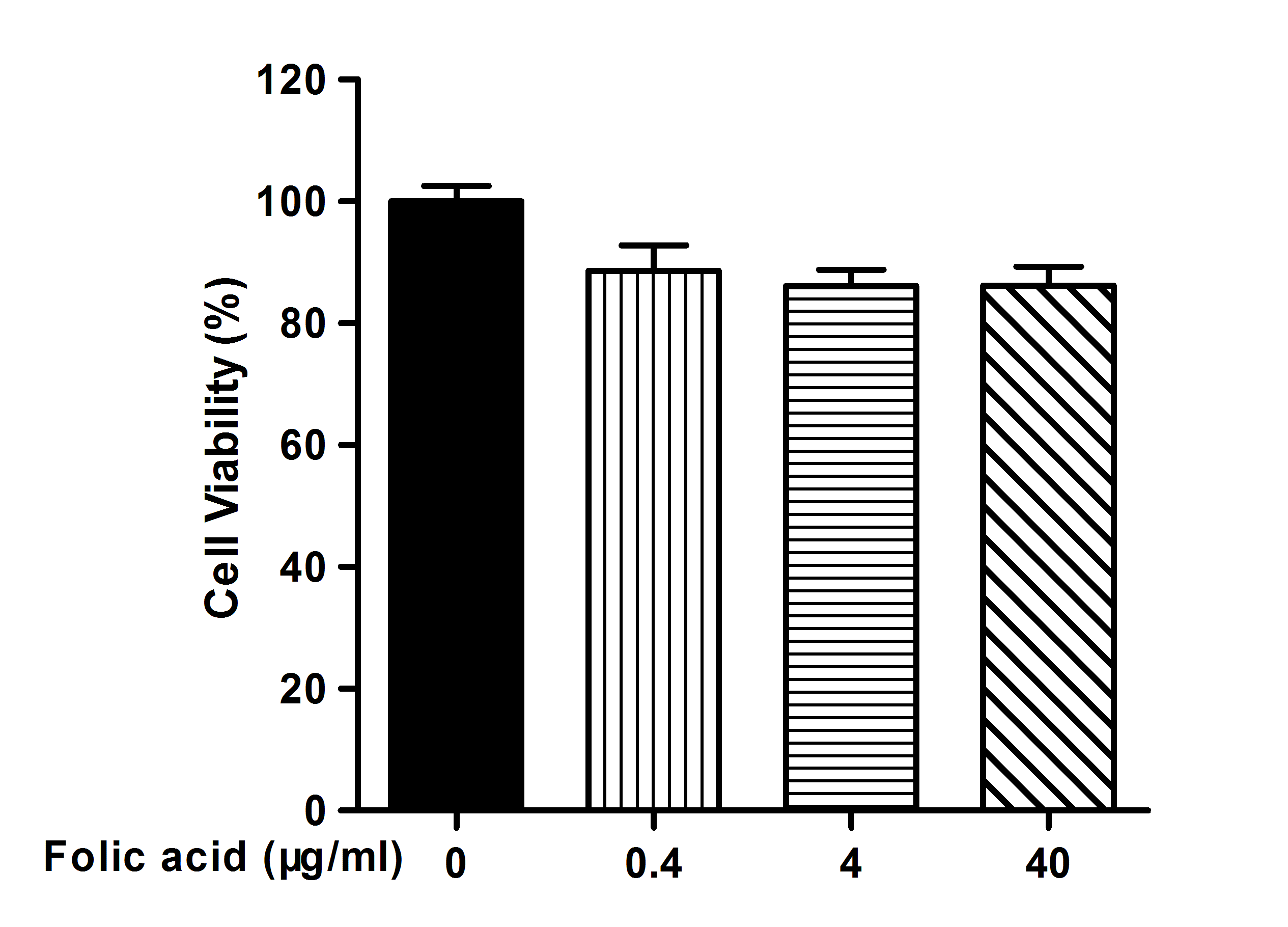

Supplement: S1 Fig — The cytotoxic effects of folic acid in THP-1 cells were assessed using the MTT assay. Data are presented as mean ± SEM from four independent experiments. (TIF) [file pone.0151553.s001.tif]
